# Corrigendum: A Practical Guide to Sparse *k*-Means Clustering for Studying Molecular Development of the Human Brain

**DOI:** 10.3389/fnins.2022.907479

**Published:** 2022-04-19

**Authors:** Justin L. Balsor, Keon Arbabi, Desmond Singh, Rachel Kwan, Jonathan Zaslavsky, Ewalina Jeyanesan, Kathryn M. Murphy

**Affiliations:** ^1^McMaster Neuroscience Graduate Program, McMaster University, Hamilton, ON, Canada; ^2^Department of Psychology, Neuroscience and Behavior, McMaster University, Hamilton, ON, Canada

**Keywords:** human brain, development, clustering, synaptic proteins, transcriptomic data, high dimension and low sample size, sparsity-based algorithm

In the original article, there was an error in the section ***Discussion, Paragraph Number 2*** **as published**. The following citations were missing (Murphy et al., 2005; Pinto et al., 2010, 2015; Williams et al., 2010; Witten and Tibshirani, 2010; Siu et al., 2015, 2017). The revised paragraph is included below:

“Many factors come into play when selecting an appropriate clustering algorithm for a study. Here, we considered the goal of the study (to resolve sometimes subtle age-related changes in molecular mechanism), the structure of the dataset (*p* ~ *n* to *p* >> *n*), and the output of the algorithm (is it just the clusters or is feature selection included). Sparse K-means clustering was selected because it fit all of those considerations. We know from previous studies of the molecular development of the human brain that there can be subtle differences between age groups (Murphy et al., 2005; Pinto et al., 2010, 2015; Williams et al., 2010; Siu et al., 2015, 2017), and yet even small changes in protein or gene expression will alter neural function. Therefore, we looked for algorithms designed for omics datasets where subtle changes in a subset of the genes or proteins would identify important characteristics of the data. The development of sparse K-means clustering by Witten and Tibshirani (2010) was partially inspired by the need to better cluster a breast cancer dataset. In that dataset, subtle differences in gene expression significantly impacted patient outcomes, but standard clustering approaches did not pick those up. In addition, sparse clustering was developed to address datasets, like ours and the breast cancer data where the structure is *p* ~ *n* to *p* >> *n*. Sparse K-means clustering is a good fit for those high dimensional structures because it minimizes the within-cluster sum of squares with a dissimilarity measure while maximizing the between-cluster sum of squares by iteratively reweighting the measures. Finally, and most importantly, sparse K-means clustering performs feature selection. The examples in this paper show the reweighted proteins and those distributions identifying how much each protein contributes to partitioning the samples into clusters. That matrix is sparse, with unimportant proteins having near-zero weights and important ones having non-zero weights. Those weights are essential for cluster analysis to help with making neurobiologically relevant interpretations of brain development from the cluster analysis.”

The authors apologize for this error and state that this does not change the scientific conclusions of the article in any way. The original article has been updated.

## Publisher's Note

All claims expressed in this article are solely those of the authors and do not necessarily represent those of their affiliated organizations, or those of the publisher, the editors and the reviewers. Any product that may be evaluated in this article, or claim that may be made by its manufacturer, is not guaranteed or endorsed by the publisher.
