# Supplementary material for: A Practical Guide to Sparse k-Means Clustering for Studying Molecular Development of the Human Brain
Source: Front Neurosci. 2021 Nov 16;15:668293. doi: 10.3389/fnins.2021.668293 (PMC8636820; doi:10.3389/fnins.2021.668293)
Supplement: Supplementary file 1 [file Data_Sheet_1.pdf]

A Practical Guide to Sparse Clustering for Studying Molecular Development of the Human Brain

Supplementary Material – Glossary of terms, Tables and Figures

Supplementary Box 1. Glossary

	Term	Definition
**1**	*Feature*	A measurable piece of the collected data, such as the expression of an individual gene or protein. The number of features in a dataset is often denoted with *p.*
**2**	*High Dimensional data*	Describes datasets where observations have been spread across many features/dimensions, and grow increasingly equidistant from one another; common when the number of features *(p)* is approximately equal to, or greater than, the number of observations *(n) (p>>n).* As dimensions are added, data become sparser in the high dimensional space.
**3**	*Dimension reduction*	Methods that aim to find features which adequately characterize the original high-dimensional data in a lower-dimensional space.
**4**	*Subspace*	A subset of features within the dataset.
**5**	*k*	A parameter that is used to specify a desired number of clusters.
**6**	*Sparsity-based clustering methods*	Clustering algorithms that use all *p* features in a dataset but either subset or reweigh features according to how well they contribute to cluster formation. These methods are further divided into '*Subspace clustering methods*' and '*Sparse k-means clustering methods*'.
**7**	*Subspace clustering methods*	An extension of traditional clustering that attempts to find clusters in different subspaces of the same dataset.
**8**	*Sparse k-means clustering methods*	An extension of traditional clustering that addresses sparsity by adaptively searching both the local and global features.
**9**	*Medoid*	The data point (x_i) within a cluster (Y) with the smallest average dissimilarity between all other data points in *Y.*
**10**	*Unsupervised*	Data-driven and independent of external classifications (e.g. age, sex, experimental condition).
**11**	*Supervised*	Dependent on external classifications (e.g. age, sex, experimental condition).
**13**	*Curse of Dimensionality*	Adding observations increases the dimensionality and as the number of observations grows, so too does the likelihood that only a subset of them will contribute to partitioning samples into clusters. As a result, a very large number of dimensions can become a *curse* because they may obscure the impact of a subset of relevant observations.

**14**	*Feature Weight*	A measure that quantifies the extent to which a feature contributes to cluster formation. Large weights denote that a feature is important to forming clusters

Supplementary Table 1. *Human samples from Murphy lab studies*. The age, age group, biological sex, and post-mortem interval (PMI) for each of the human cortical tissue samples is listed.

Age	Age Group	Sex	PMI (Hours)
20 days	Neonate	M	9
86 days	Neonate	F	23
96 days	Neonate	M	12
98 days	Neonate	M	16
119 days	Neonate	M	22
120 days	Neonate	M	23
133 days	Infant	M	16
136 days	Infant	F	11
273 days	Infant	M	10
1 year 123 days	Young Children	M	21
2 years 57 days	Young Children	F	21
2 years 75 days	Young Children	F	11
3 years 123 days	Young Children	F	11
4 years 203 days	Young Children	M	15
4 years 258 days	Young Children	M	17
5 years 144 days	Older Children	M	17
8 years 50 days	Older Children	F	20
8 years 214 days	Older Children	F	20
9 years 46 days	Older Children	F	20
12 years 164 days	Teens	M	22
13 years 99 days	Teens	M	5
15 years 81 days	Teens	M	16
19 years 76 days	Teens	F	16
22 years 359 days	Young Adults	M	4
32 years 223 days	Young Adults	M	13
50 years 156 days	Young Adults	M	8
53 years 330 days	Young Adults	F	5
69 years 110 days	Older Adults	M	12
71 years 333 days	Older Adults	F	9
79 years 181 days	Older Adults	F	14

Supplementary Table 2. *Human samples from Kang et al. (2011)*. Samples for the primary visual cortex (V1C) are shown. Age is either postnatal months (PMonth) or years (Y).

ID	Brain Code	Region	Sex	Age	Years	Weeks	PMI	Hemisphere
GSM704398	HSB121	V1C	M	4 PMonth	0.33	17	9.5	L
GSM704399	HSB121	V1C	M	4 PMonth	0.33	17	9.5	R
GSM704607	HSB132	V1C	M	4 PMonth	0.33	17	22	L
GSM704608	HSB132	V1C	M	4 PMonth	0.33	17	22	R
GSM704729	HSB139	V1C	M	4 PMonth	0.33	17	20	L
GSM704577	HSB131	V1C	F	6 PMonth	0.5	26	26	L
GSM705106	HSB171	V1C	M	10 PMonth	0.83	43	18	L
GSM704430	HSB122	V1C	F	1 Y	1	52	18	L
GSM704431	HSB122	V1C	F	1 Y	1	52	18	R
GSM704802	HSB143	V1C	F	2 Y	2	104	12	L
GSM704803	HSB143	V1C	F	2 Y	2	104	12	R
GSM705120	HSB172	V1C	M	3 Y	3	156	16	L
GSM705133	HSB173	V1C	F	3 Y	3	156	8	L
GSM704353	HSB118	V1C	M	4 Y	4	208	20	R
GSM704745	HSB141	V1C	M	8 Y	8	416	30	L
GSM705145	HSB174	V1C	M	8 Y	8	416	16	L
GSM705159	HSB175	V1C	F	11 Y	11	572	22	L
GSM704492	HSB124	V1C	F	13 Y	13	676	19.5	R
GSM704368	HSB119	V1C	M	15 Y	15	780	14.5	L
GSM704151	HSB105	V1C	M	18 Y	18	936	28	L
GSM704152	HSB105	V1C	M	18 Y	18	936	28	R
GSM704545	HSB127	V1C	F	19 Y	19	988	9.5	L
GSM704561	HSB130	V1C	F	21 Y	21	1092	18	L
GSM704775	HSB142	V1C	M	22 Y	22	1144	18	L
GSM704776	HSB142	V1C	M	22 Y	22	1144	18	R
GSM704711	HSB136	V1C	M	23 Y	23	1196	10.5	L
GSM704712	HSB136	V1C	M	23 Y	23	1196	10.5	R
GSM704619	HSB133	V1C	F	27 Y	27	1404	21	L
GSM704527	HSB126	V1C	F	30 Y	30	1560	9.5	L
GSM704528	HSB126	V1C	F	30 Y	30	1560	9.5	R
GSM704866	HSB145	V1C	M	36 Y	36	1872	18	L
GSM704867	HSB145	V1C	M	36 Y	36	1872	18	R
GSM704461	HSB123	V1C	M	37 Y	37	1924	13	L
GSM704462	HSB123	V1C	M	37 Y	37	1924	13	R

GSM705253	HSB187	V1C	F	37 Y	37	1924	10	L
GSM705254	HSB187	V1C	F	37 Y	37	1924	10	R
GSM704664	HSB135	V1C	F	40 Y	40	2080	30.5	L
GSM704666	HSB135	V1C	F	40 Y	40	2080	30.5	R
GSM704834	HSB144	V1C	M	40 Y	40	2080	28	L
GSM704835	HSB144	V1C	M	40 Y	40	2080	28	R
GSM705214	HSB183	V1C	M	42 Y	42	2184	19	L
GSM705198	HSB182	V1C	M	55 Y	55	2860	13	L
GSM704183	HSB106	V1C	M	64 Y	64	3328	4	L
GSM704184	HSB106	V1C	M	64 Y	64	3328	4	R
GSM704246	HSB111	V1C	F	70 Y	70	3640	13	L
GSM704247	HSB111	V1C	F	70 Y	70	3640	13	R
GSM705062	HSB156	V1C	F	82 Y	82	4264	16	L
GSM705063	HSB156	V1C	F	82 Y	82	4264	16	R

Supplementary Table 3. *List of 23 proteins measured by Western blotting*. The antibodies, concentrations, suppliers, and RRIDs are listed. The citations for the publications are also included.

Protein	Antibody Concentration and Supplier	Citation
GAD65 (glutamic acid decarboxylase 65)	1:500 (Chemicon International, Temecula, CA, USA)	Pinto et al. (2010)
GAD67 (glutamic acid decarboxylase 67)	1:1000 (Chemicon International, Temecula, CA, USA)	
VGAT	1:1000 (Synaptic Systems, Göttingen, Germany)	
$GABA_A\alpha1$	1:500 (Imgenex, San Diego, CA, USA)	
$GABA_A\alpha2$	1:1000 (Imgenex, San Diego, CA, USA)	
$GABA_A\alpha3$	1:1000 (Imgenex, San Diego, CA, USA)	
CB1	1:1000 (Cayman, Ann Arbor, MI, USA)	
Gephyrin	1:500 (Chemicon International, Temecula, CA, USA)	
Gephyrin Homogenate	1:2000 (Millipore, Billerica, MA)	Pinto et al. (2015)
PSD95 Homogenate	1:32000 (Millipore, Billerica, MA)	
Synapsin	1:8000 (Invitrogen, Carlsbad, CA)	
Synaptophysin	1:2000 (Sigma-Aldrich, St. Louis, MO)	
GluA2	1:1000 (RRID: AB_2533058, Invitrogen)	Siu et al. (2017)
GluN1/NR1	1:4000 (RRID: AB_396353, BD Biosciences PharMingen)	
GluN2A/NR2A	1:1000 (RRID: AB_95169, EMD Millipore)	
GluN2B/NR2B	1:1000 (RRID: AB_2112925, EMD Millipore)	
PSD95	1:16000 (RRID: AB_94278, EMD Millipore)	
Golli-MBP	1:4000 [AB62631] (Abcam, Cambridge, MA, USA)	Siu et al. (2015)
Classic-MBP	1:4000 [AB62631] (Abcam, Cambridge, MA, USA)	
Ube3A (E6AP)	1:1000 (Bethyl Laboratories, Montgomery, TX, USA)	Williams et al. (2010)
Integrinβ3	1:1000 [AB2984] (Millipore, Billerica, MA, USA)	Unpublished
GFAP	1:2000 [MAB360] (Millipore, Billerica, MA, USA)	
Drebrin	1:1000 [10R-D117A] (Fitzgerald Industries International, Acton, MA, USA)	

Supplementary Table 4. *List of the 95 proteins, including their gene symbol and short name.*

Gene Symbol	Protein Name	Short Name
ALCAM	CD166 antigen	
ANG	Angiogenin	
AXL	Tyrosine-protein kinase receptor UFO	
CCL14	C-C motif chemokine 14	
CCL18	C-C motif chemokine 18	
CCL2	C-C motif chemokine 2	
CCL5	C-C motif chemokine 5	
CD14	Monocyte differentiation antigen CD14	
CEACAM1	Carcinoembryonic antigen-related cell adhesion molecule 1	
CNTN2	Contactin-2	
CSF1	Macrophage colony-stimulating factor 1	CSF-1
CSF1R	Macrophage colony-stimulating factor 1 receptor	
CTSS	Cathepsin S	
CXCL11	C-X-C motif chemokine 11	
CXCL16	C-X-C motif chemokine 16	
CXCL8	Interleukin-8	IL-8
EGFR	Epidermal growth factor receptor	
ERBB3	Receptor tyrosine-protein kinase erbB-3	
FAS	Tumor necrosis factor receptor superfamily member 6	
FCGR2B	Low affinity immunoglobulin gamma Fc region receptor II-b	IgG Fc receptor II-b
FGF2	Fibroblast growth factor 2	FGF-2
FLT1	Vascular endothelial growth factor receptor 1	VEGFR-1
FLT3LG	Fms-related tyrosine kinase 3 ligand	Flt3 ligand
GDF15	Growth/differentiation factor 15	GDF-15
GDNF	Glial cell line-derived neurotrophic factor	hGDNF
HGF	Hepatocyte growth factor	
ICAM1	Intercellular adhesion molecule 1	ICAM-1
ICAM2	Intercellular adhesion molecule 2	ICAM-2
IGFBP1	Insulin-like growth factor-binding protein 1	IBP-1
IGFBP2	Insulin-like growth factor-binding protein 2	IBP-2
IGFBP6	Insulin-like growth factor-binding protein 6	IBP-6
IL11	Interleukin-11	IL-11
IL13RA1	Interleukin-13 receptor subunit alpha-1	IL-13 receptor subunit alpha-1

IL13RA2	Interleukin-13 receptor subunit alpha-2	IL-13 receptor subunit alpha-2
IL16	Pro-interleukin-16	
IL1A	Interleukin-1 alpha	IL-1 alpha
IL1RN	Interleukin-1 receptor antagonist protein	IL-1RN
IL2RB	Interleukin-2 receptor subunit beta	IL-2 receptor subunit beta
IL2RG	Cytokine receptor common subunit gamma	
IL4	Interleukin-4	IL-4
IL6R	Interleukin-6 receptor subunit alpha	IL-6 receptor subunit alpha
IL6ST	Interleukin-6 receptor subunit beta	IL-6 receptor subunit beta
IL9	Interleukin-9	IL-9
KIT	Mast/stem cell growth factor receptor Kit	SCFR
LCN2	Neutrophil gelatinase-associated lipocalin	NGAL
LIF	Leukemia inhibitory factor	LIF
LYVE1	Lymphatic vessel endothelial hyaluronic acid receptor 1	LYVE-1
MICA	MHC class I polypeptide-related sequence A	MIC-A
NRCAM	Neuronal cell adhesion molecule	Nr-CAM
PDGFA	Platelet-derived growth factor subunit A	PDGF subunit A
PDGFAB	Platelet-derived growth factor subunit AB	PDGF subunit AB
PDGFB	Platelet-derived growth factor subunit B	PDGF subunit B
PECAM1	Platelet endothelial cell adhesion molecule	PECAM-1
PF4	Platelet factor 4	PF-4
PI3	Elafin	
PLAUR	Urokinase plasminogen activator surface receptor	U-PAR
PPBP	Platelet basic protein	PBP
PROK1	Prokineticin-1	
RETN	Resistin	
SCARB2	Lysosome membrane protein 2	
SIGLEC5	Sialic acid-binding Ig-like lectin 5	Siglec-5
SPP1	Osteopontin	
TGFB1	Human TGF-beta 1 cDNA	
TGFA	Protransforming growth factor alpha	
TIMP1	Metalloproteinase inhibitor 1	
TIMP2	Metalloproteinase inhibitor 2	
TNF	Tumor necrosis factor	
TNFRSF10C	Tumor necrosis factor receptor superfamily member 10C	

TNFRSF14	Tumor necrosis factor receptor superfamily member 14	
TNFRSF1A	Tumor necrosis factor receptor superfamily member 1A	
TNFRSF1B	Tumor necrosis factor receptor superfamily member 1B	
TYRO3	Tyrosine-protein kinase receptor TYRO3	
CNR1	Cannabinoid receptor 1	CB-R
MBP	Classic myelin basic protein	MBP
DBN1	Drebrin	
GABRA1	Gamma-aminobutyric acid receptor subunit alpha-1	
GABRA2	Gamma-aminobutyric acid receptor subunit alpha-2	
GABRA3	Gamma-aminobutyric acid receptor subunit alpha-3	
GAD2	Glutamate decarboxylase 2	
GAD1	Glutamate decarboxylase 1	
GPHN	Gephyrin	
GPHN	Gephyrin (HOM)	
GFAP	Glial fibrillary acidic protein	GFAP
GRIA2	Glutamate receptor 2	GluR-2
GRIN1	Glutamate receptor ionotropic, NMDA 1	GluN1
MBP	Golli myelin basic protein	Golli MBP
ITGB3	Integrin beta-3	
GRIN2A	Glutamate receptor ionotropic, NMDA 2A	GluN2A
GRIN2B	Glutamate receptor ionotropic, NMDA 2B	GluN2B
DLG4	Disks large homolog 4	PSD-95
DLG4	Disks large homolog 4 (HOM)	PSD-95
SYN1	Synapsin-1	NA
SYP	Synaptophysin	NA
UBE3A	Ubiquitin-protein ligase E3A	NA
SLC32A1	Vesicular inhibitory amino acid transporter	hVIAAT

Supplementary Table S5: Summary of descriptive statistics and 95% confidence intervals for clusters produced by 100 iterations of RSKC on 23 proteins.

Cluster	Descriptive Statistics			95% Confidence Interval	
	**Count**	**Mean**	**SD**	**Lower Limit**	**Upper Limit**
A	819	1.25	2.65	1.06	1.43
B	263	2.76	4.22	2.25	3.27
C	276	7.13	9.63	5.99	8.26
D	463	10.33	8.43	9.56	11.09
E	569	15.08	10.60	14.21	15.95
F	610	52.88	26.39	50.79	54.98

Supplementary Table S6. List of 13 features (protein sums and indices), corresponding formulae, and reported source.

Feature	Formula	Source
Protein Sum ($\cos^2 > 0.5$)	$\frac{(PSD95 + Gephyrin + PSD95HOM + GFAP + Golli + GluN1 + CB1 + Classic + Synapsin + VGAT + GluA2 + Gaba2 + Ube3A + NR2B + GephyrinHOM)}{15}$	Balsor (2019)
PGSS Sum	$\frac{(PSD95HOM + GephyrinHOM + Synapsin\ I + Synaptophysin)}{4}$	Pinto et al. (2015)
PSD95 and Gephyrin Sum	$\frac{PSD95 + Gephyrin}{2}$	Balsor (2019)
Drebrin and PSD95 Sum	$\frac{Drebrin + PSD95}{2}$	Novel feature
CB1 and VGAT Sum	$\frac{CB1 + VGAT}{2}$	Novel feature
NR2A:GluA2 Index	$\frac{NR2A - GluA2}{NR2A + GluA2}$	Balsor (2019)
GluA2:GluN1 Index	$\frac{GluA2 - GluN1}{GluA2 + GluN1}$	Siu et al. (2017)
Classic:Golli MBP Index	$\frac{ClassicMBP - GolliMBP}{ClassicMBP + GolliMBP}$	Siu et al. (2015)
NR2A:NR2B Index	$\frac{NR2A - NR2B}{NR2A + NR2B}$	Siu et al. (2017)
Gaba1:Gaba2 Index	$\frac{Gaba1 - Gaba2}{Gaba1 + Gaba2}$	Pinto et al. (2010)
Integrin:GFAP Index	$\frac{Integrin - GFAP}{Integrin + GFAP}$	Balsor (2019)
PSD95:Gephyrin Index	$\frac{PSD95 - Gephyrin}{PSD95 + Gephyrin}$	Pinto et al. (2010), Williams et al. (2010), Siu et al. (2017)
GluA2:Ube3A Index	$\frac{GluA2 - Ube3A}{GluA2 + Ube3A}$	Novel feature

Supplementary Figure S1. Clusters when the age of the samples are randomized.

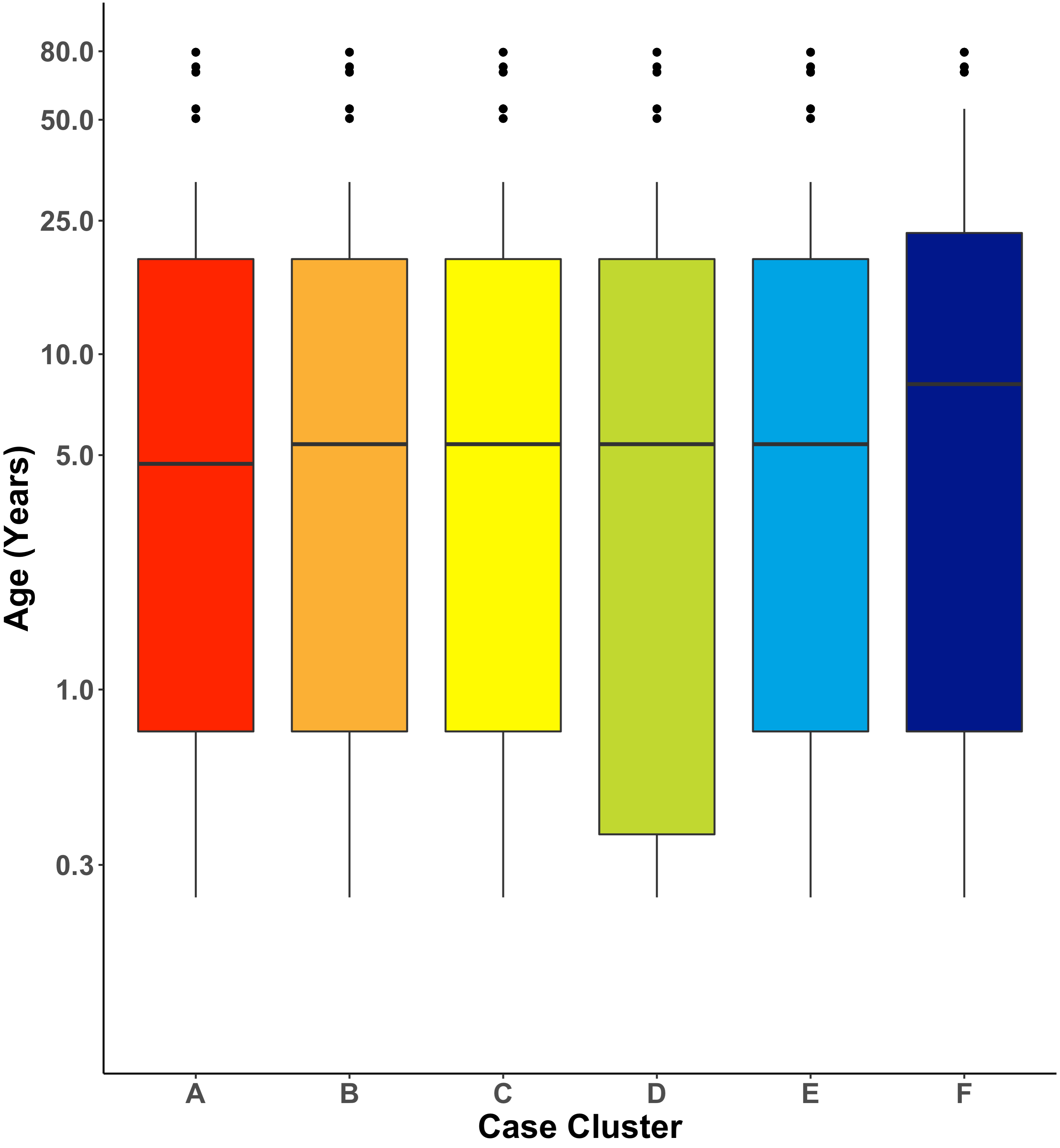

Supplementary Figure S2. Normalized expression and age binned for the 88 gene and protein pairs.

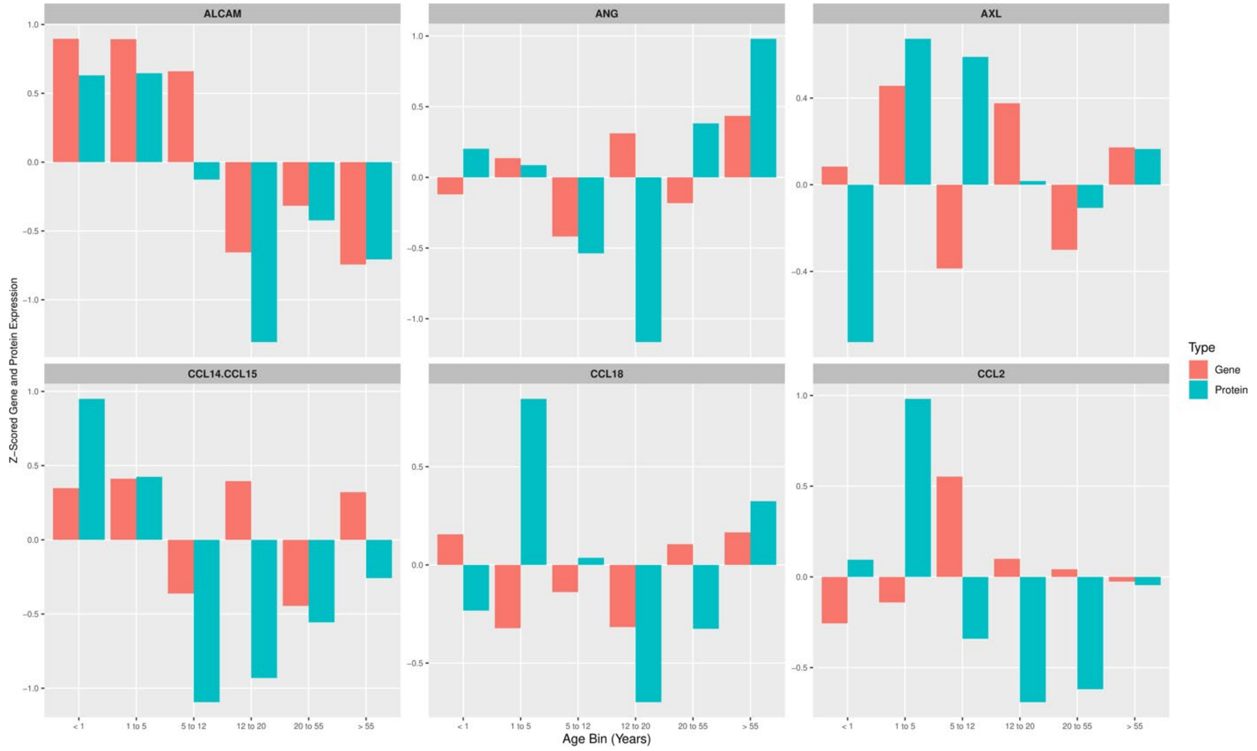

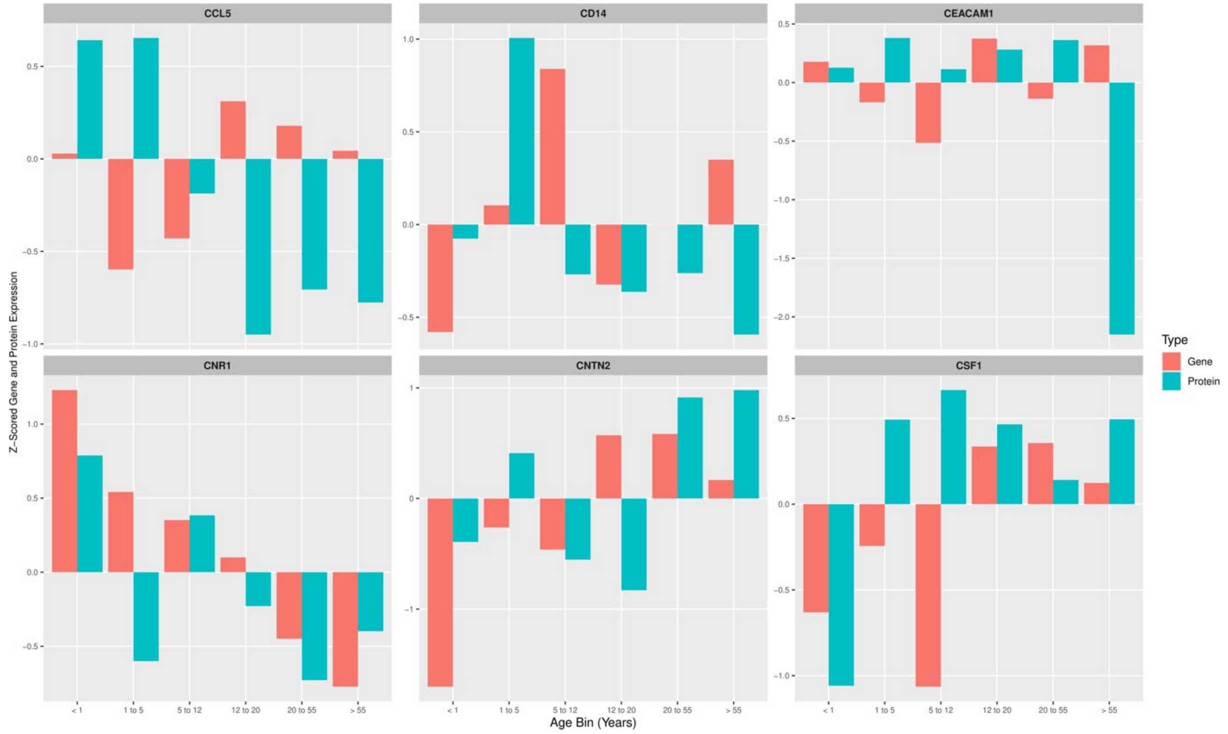

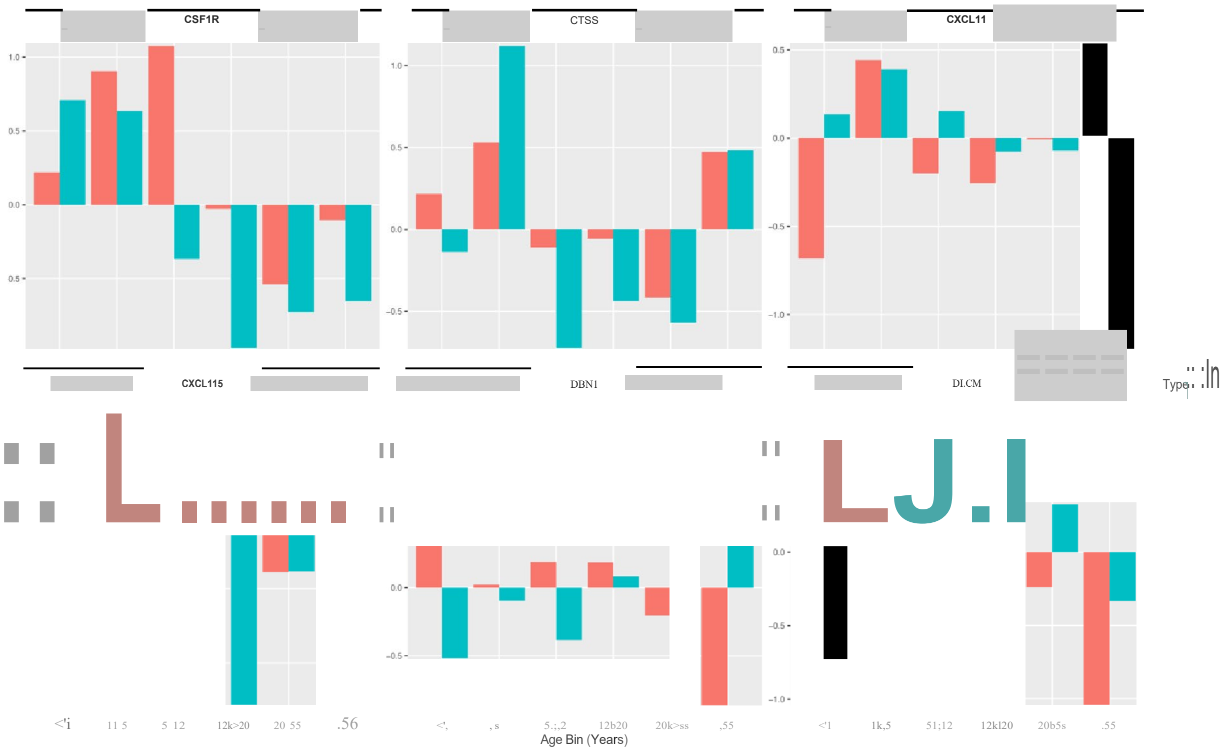
CSF1R
CTSS
CXCL11
DBN1
Age Bin (Years)
Type

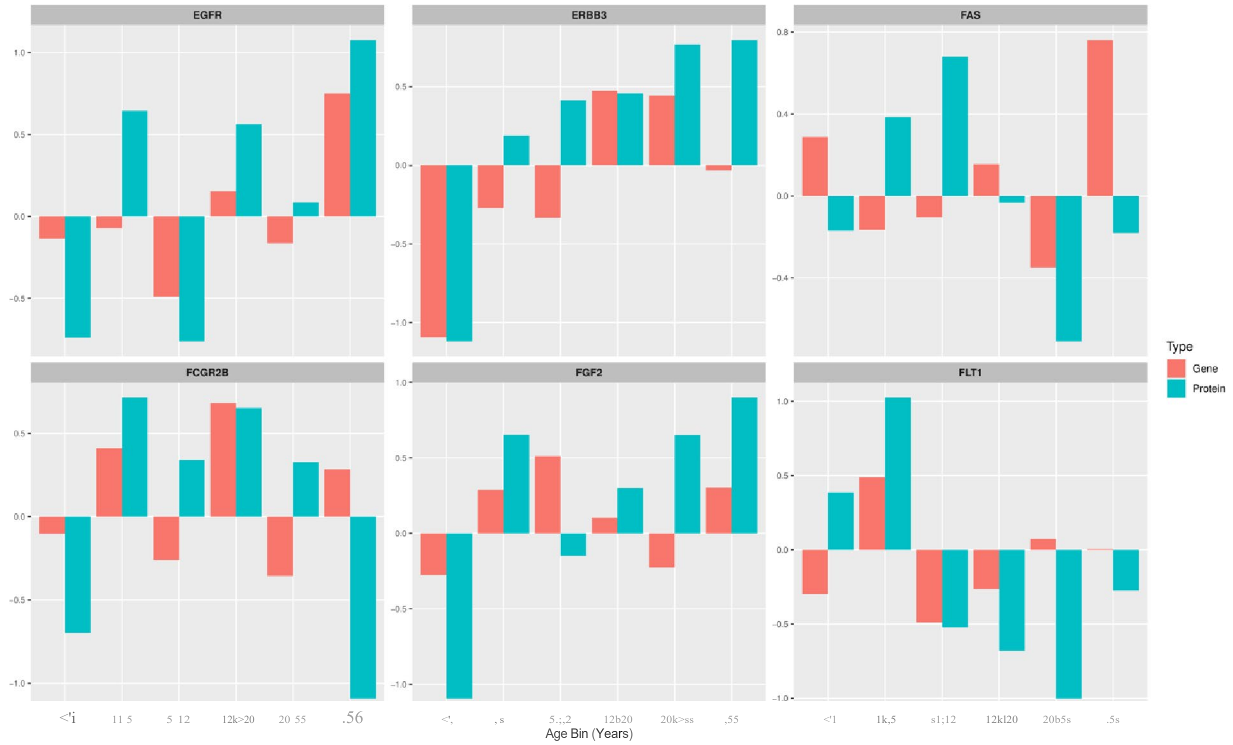
EGFR
ERBB3
FAS
FCGR2B
FGF2
FLT1
Age Bin (Years)
Type
Gene
Protein

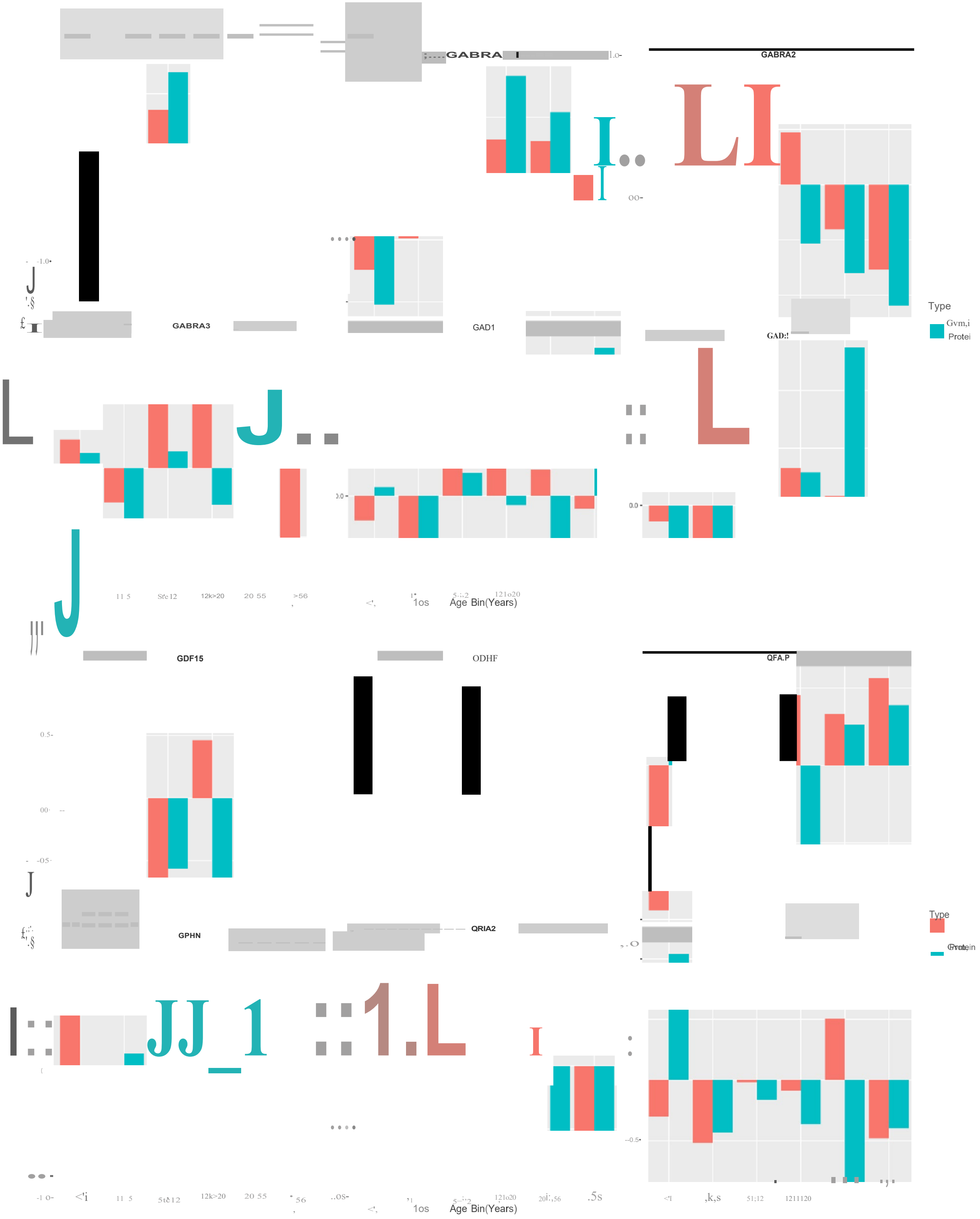
GABRA2
GABRA3
GAD1
GDF15
GPHN
Type
Age Bin(Years)

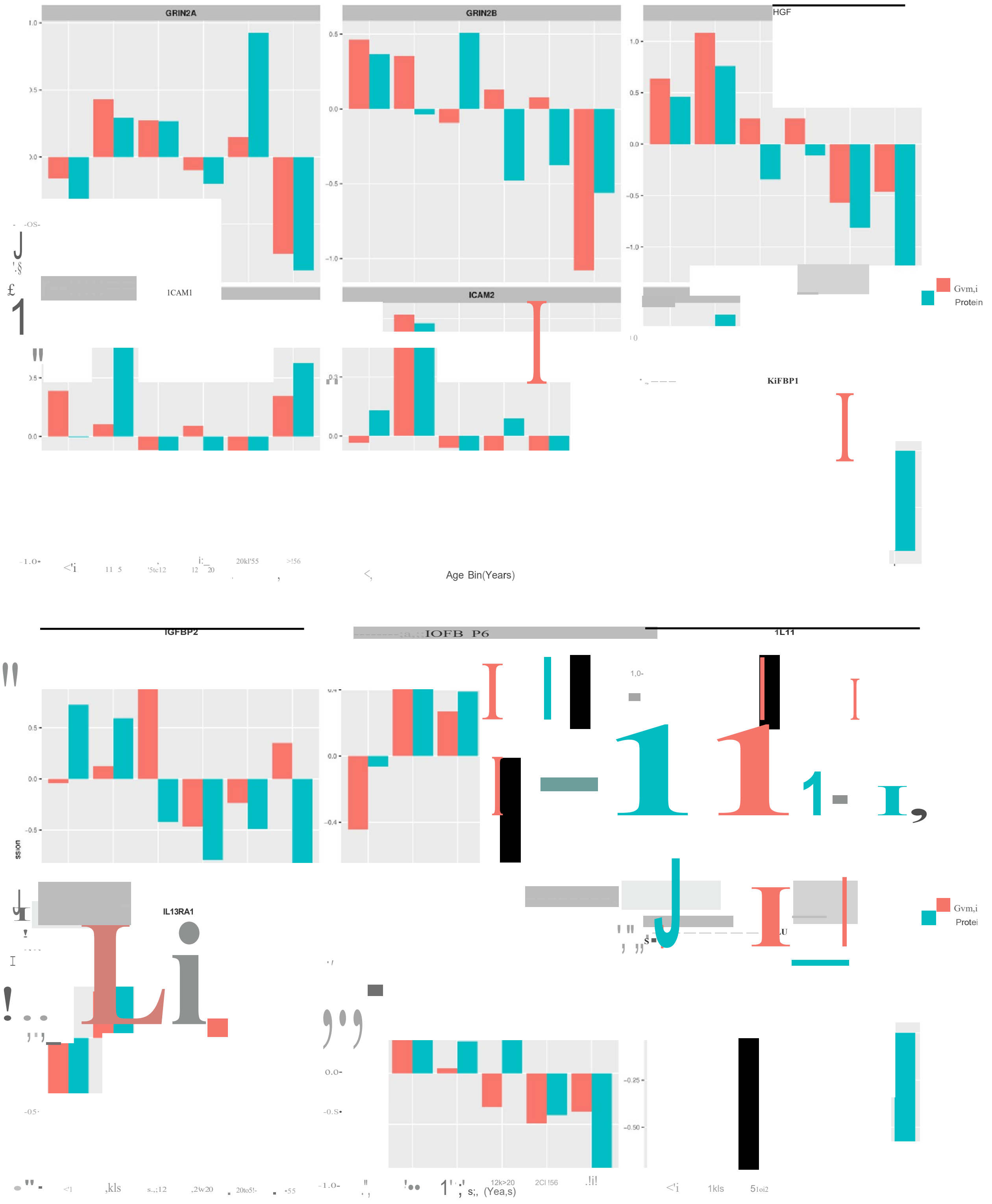
GRIN2A
GRIN2B
HGF
ICAM2
Protein
Age Bin(Years)
IGFBP2
IL13RA1

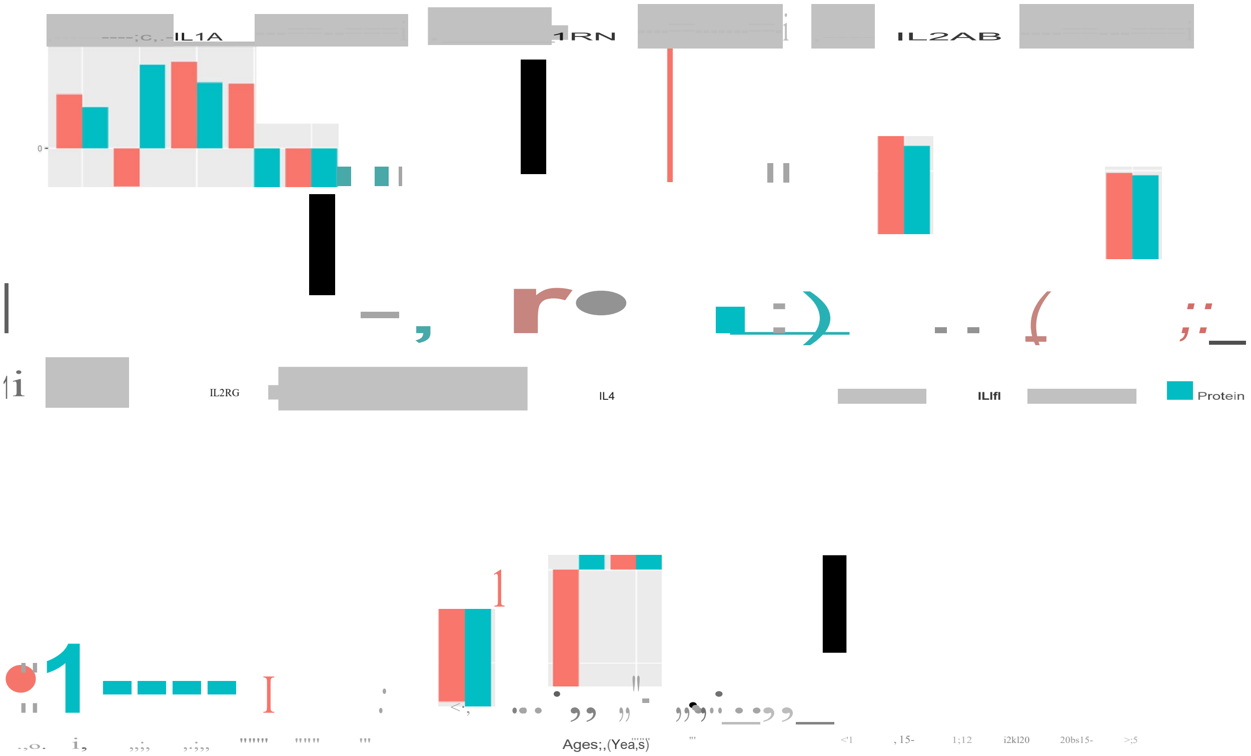
IL1A
IL2AB
IL2RG
IL4
Protein

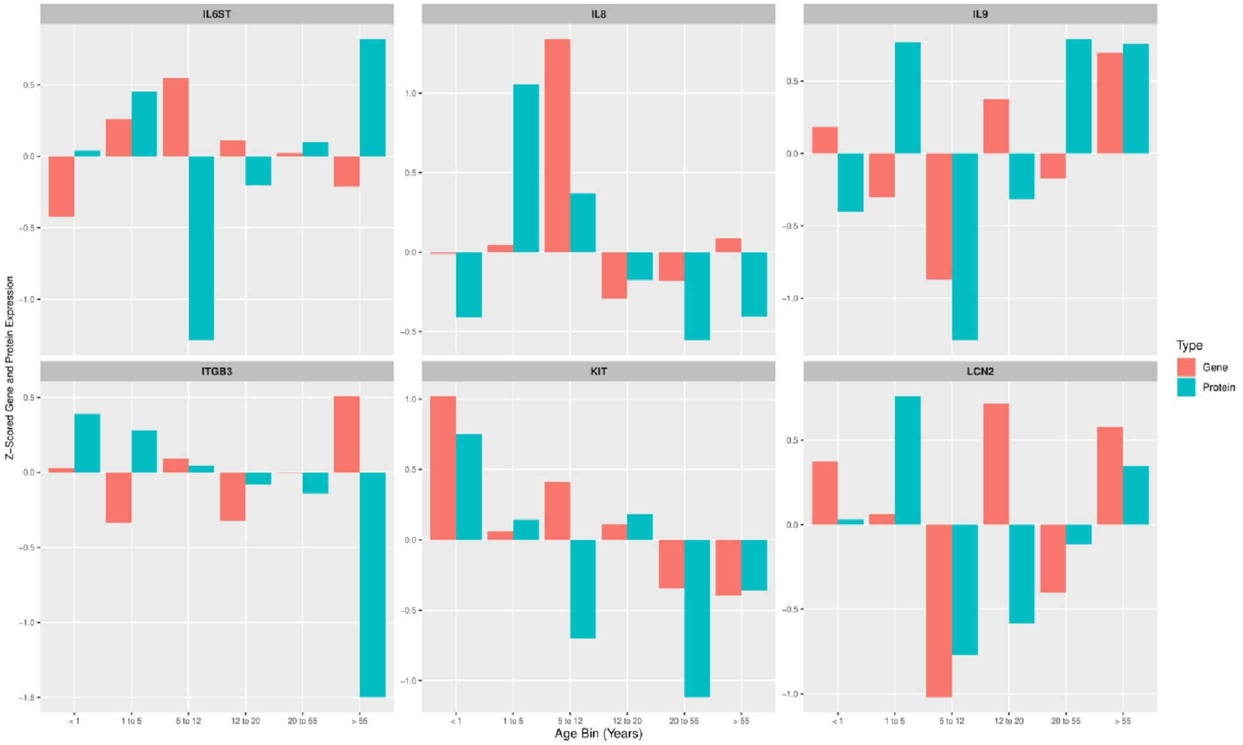
IL6ST
IL8
IL9
ITGB3
KIT
LCN2
Z-Scored Gene and Protein Expression
Age Bin (Years)
< 1
1 to 5
5 to 12
12 to 20
20 to 55
> 55
Type
Gene
Protein

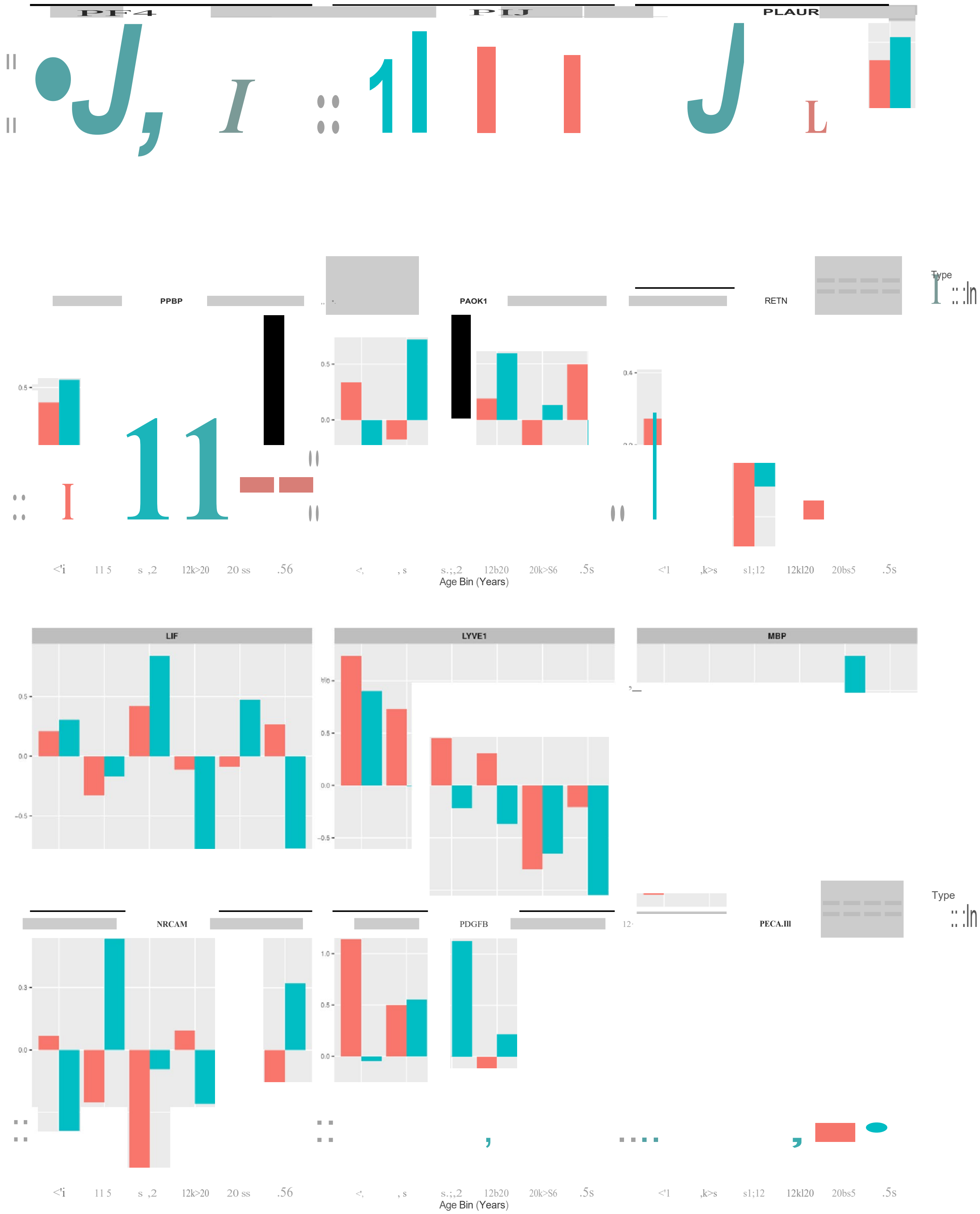
PLAUR
PPBP
RETN
Type
Age Bin (Years)
LIF
LYVE1
MBP
NRCAM
PDGFB
Type
Age Bin (Years)

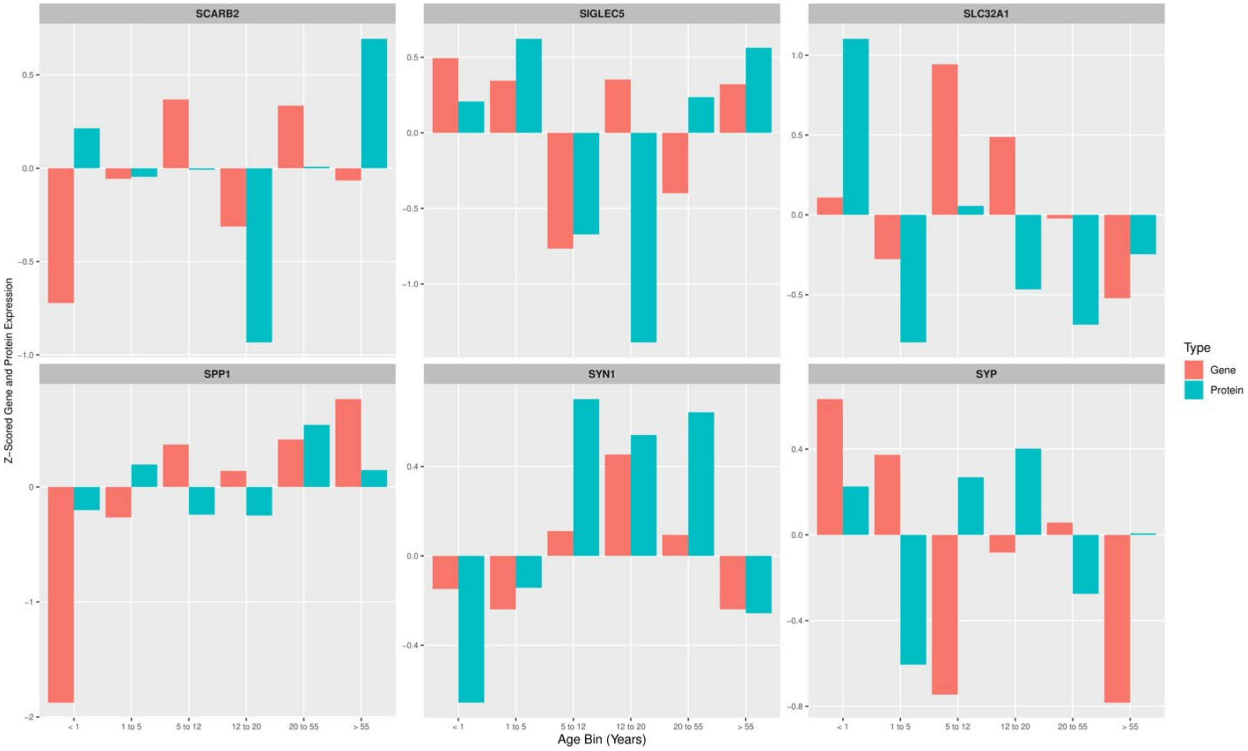
SCARB2
SIGLEC5
SLC32A1
SPP1
SYN1
SYP
Z-Scored Gene and Protein Expression
Age Bin (Years)
< 1
1 to 5
5 to 12
12 to 20
20 to 55
> 55
Type
Gene
Protein

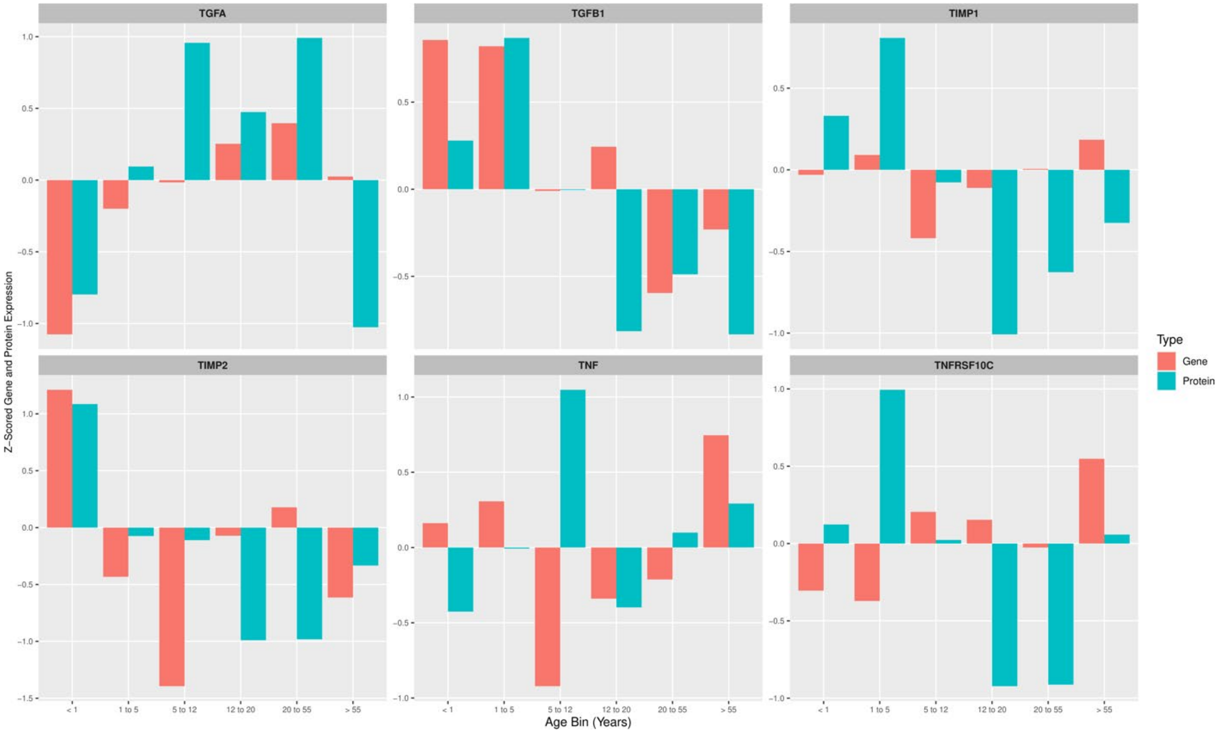
TGFA
TGFB1
TIMP1
TIMP2
TNF
TNFRSF10C
Z-Scored Gene and Protein Expression
Age Bin (Years)
< 1
1 to 5
5 to 12
12 to 20
20 to 55
> 55
Type
Gene
Protein

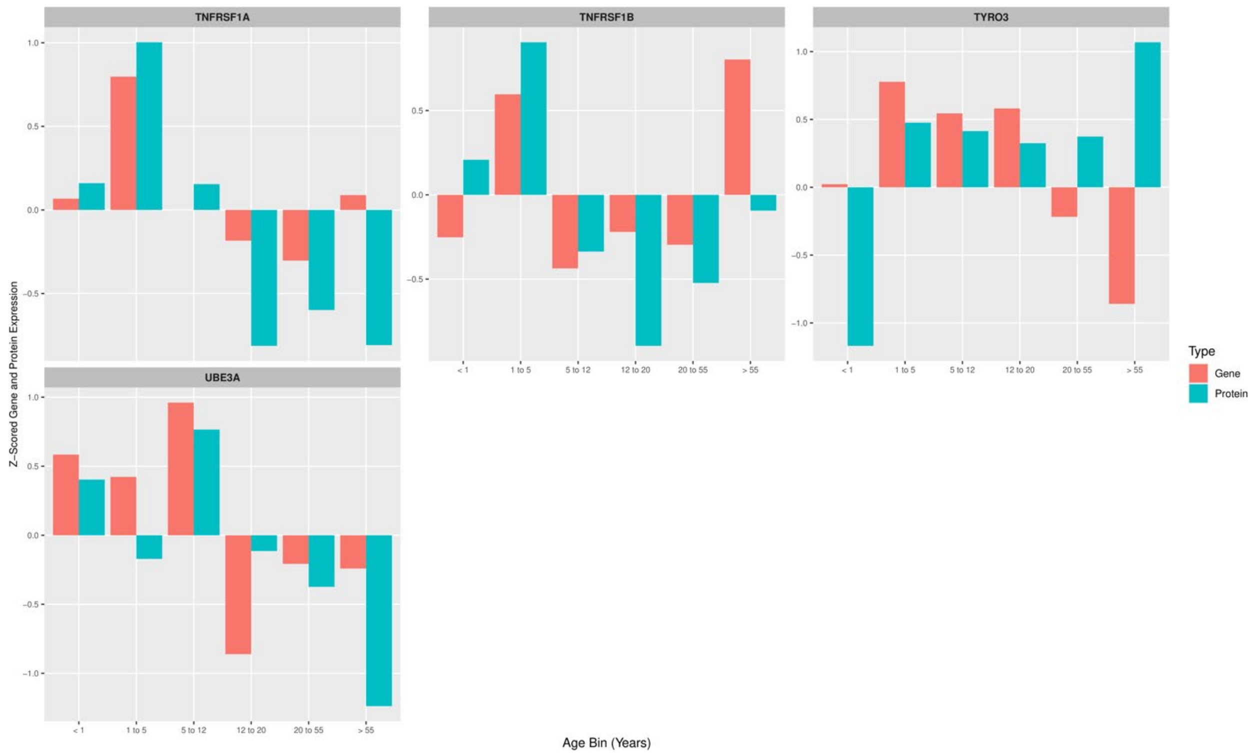

Supplementary Figure S2. *Bar plots of normalized expression for 88 gene and protein pairs across age bins*. Individual facet plots are labelled by the corresponding gene. Gene expression is represented by red bars and protein expression is shown with blue bars.

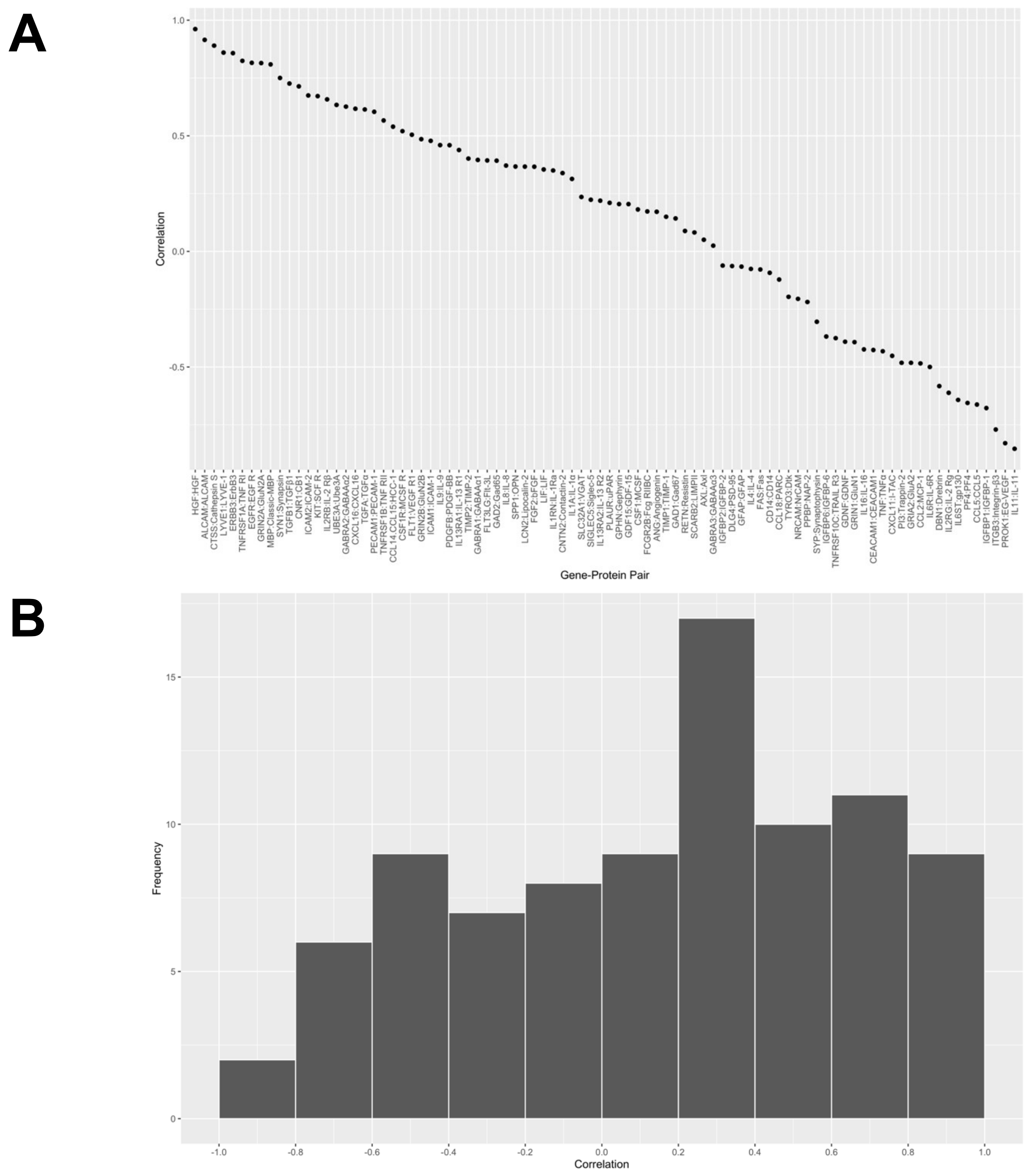

Supplementary Figure S3. **A)** *Scatterplot of the correlations obtained between corresponding gene and protein pairs*. Points represent Pearson's R correlation coefficients calculated for the expression of each of 88 gene-protein pairs, arranged in decreasing order. **B)** *Histogram of correlations obtained from expression of 88 gene-protein pairs*. Bins for the correlation are shown in increments of 0.2.

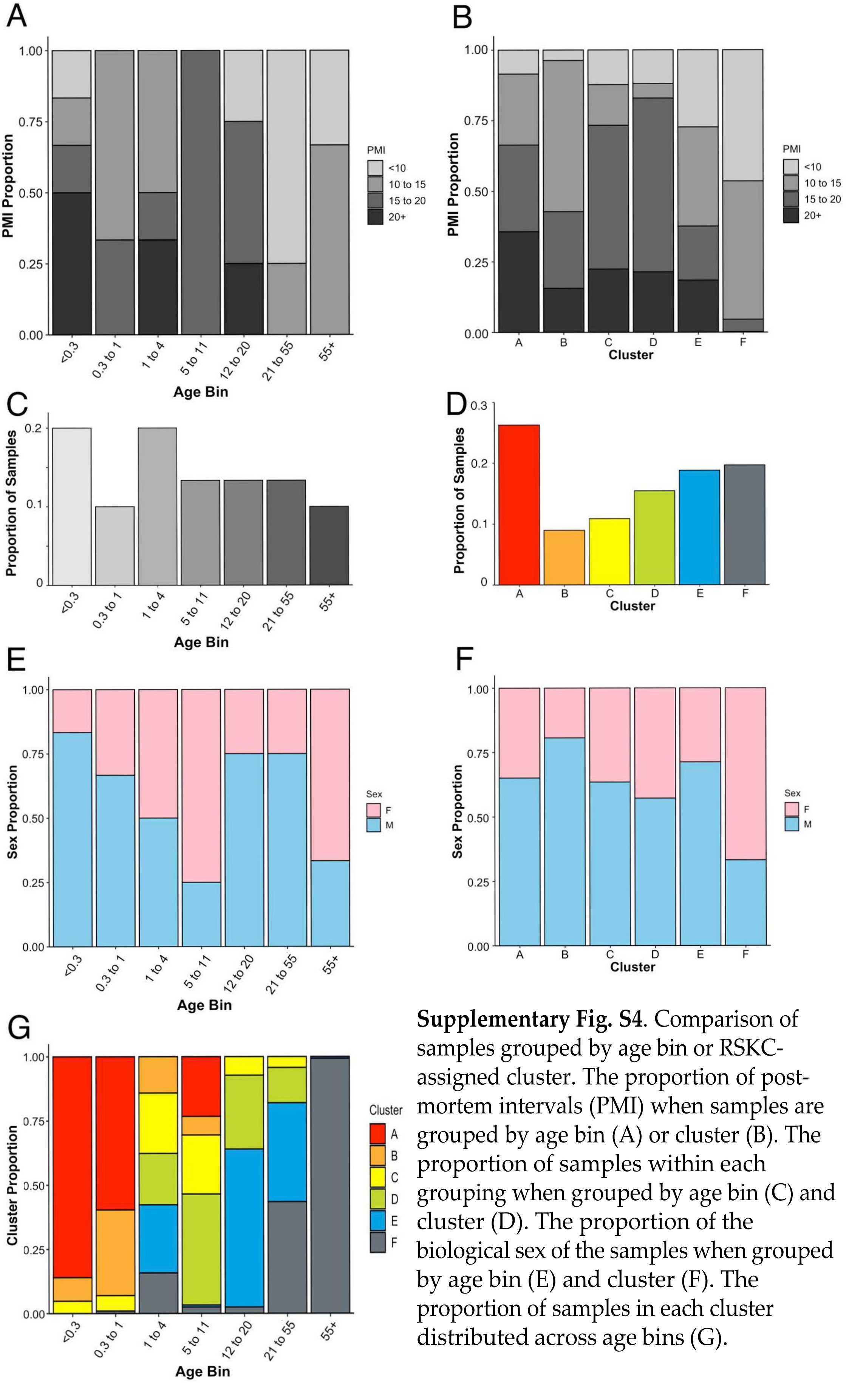

Supplementary Fig. S4. Comparison of samples grouped by age bin or RSKC-assigned cluster. The proportion of post-mortem intervals (PMI) when samples are grouped by age bin (A) or cluster (B). The proportion of samples within each grouping when grouped by age bin (C) and cluster (D). The proportion of the biological sex of the samples when grouped by age bin (E) and cluster (F). The proportion of samples in each cluster distributed across age bins (G).

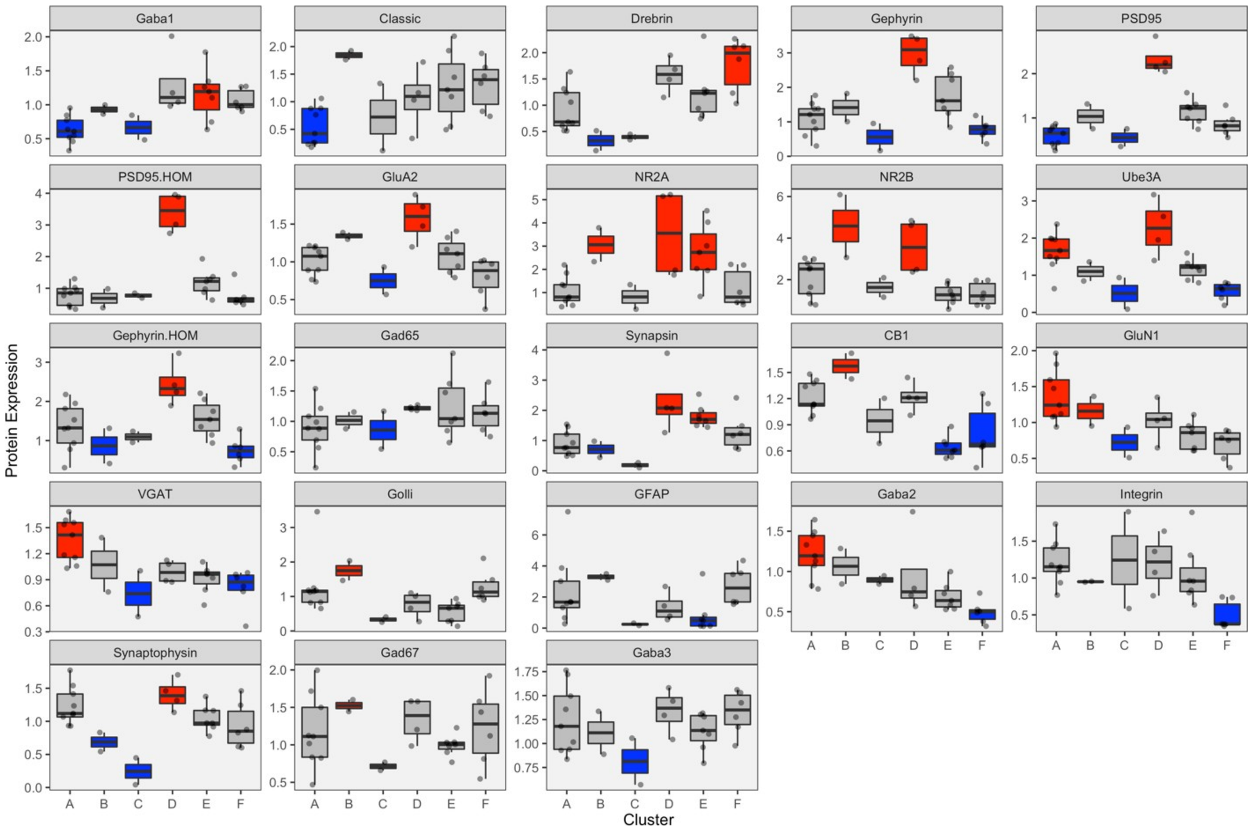

Supplementary Figure S5. *Boxplots show the expression of each protein within each cluster, with an alternate rule for identifying over- or under-representation*. Red coloured boxes represent the median expression of a group being greater than the upper quantile (i.e. 75th percentile) of all the data. Blue coloured boxes represent the median expression of a group being less than the bottom quantile (i.e. 25th percentile) of all the data. Boxes are grey if their median expression value is between the lower and upper quantiles.

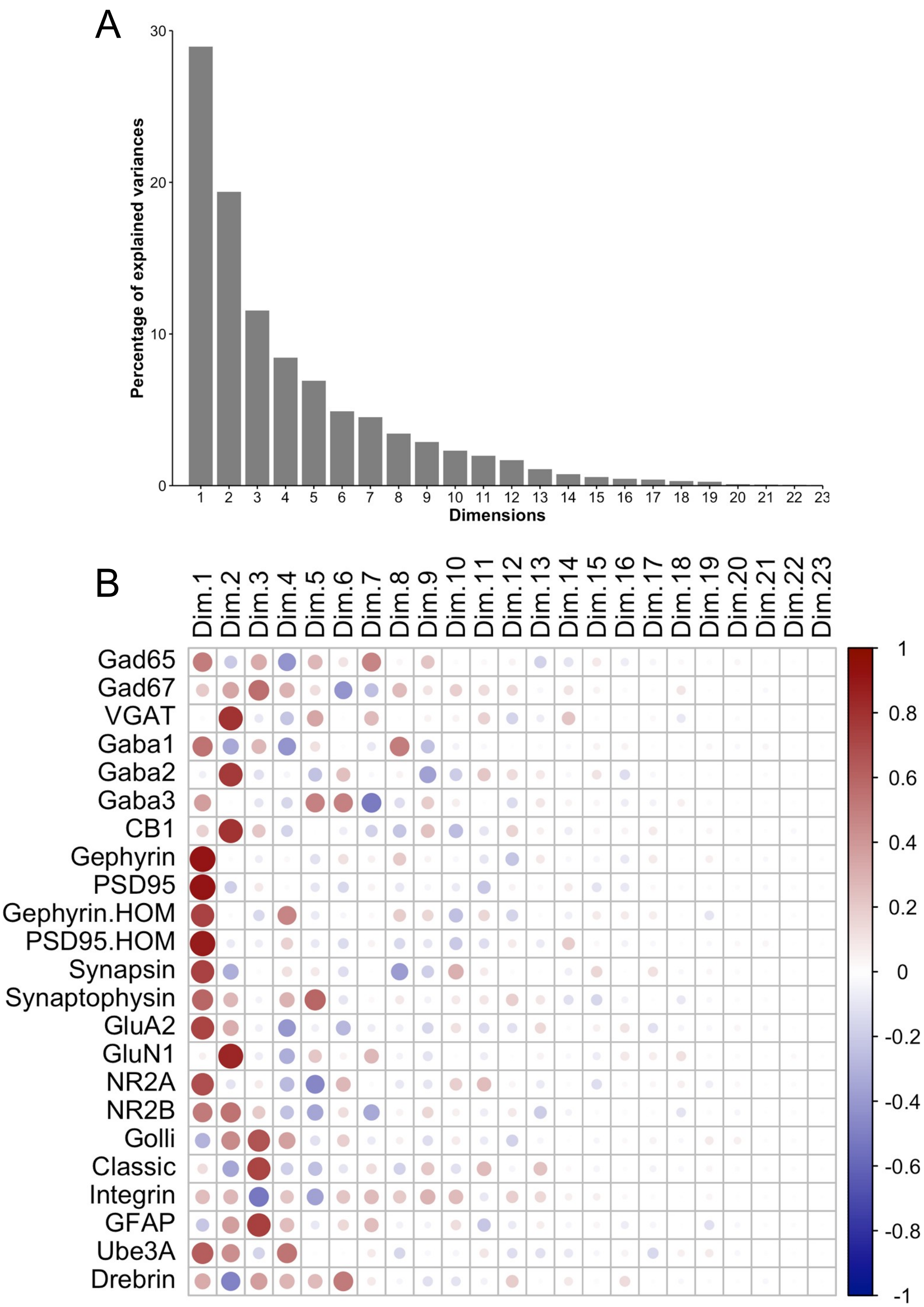

Supplementary Fig. S6. Principal component analysis to identify relevant dimensions. A) Scree plot showing the percent of explained variance captured by each principal component after the singular value decomposition (SVD). The first three principal components capture ~60% of the variance. B) Correlation matrix of esch protein with all 23 PCA dimensions. The strength of the correlation is represented by the circle size (large =strong, small= weak) while the direction is represented by the circle colour (negative = blue, positive = red) of Pearson's R correlation.

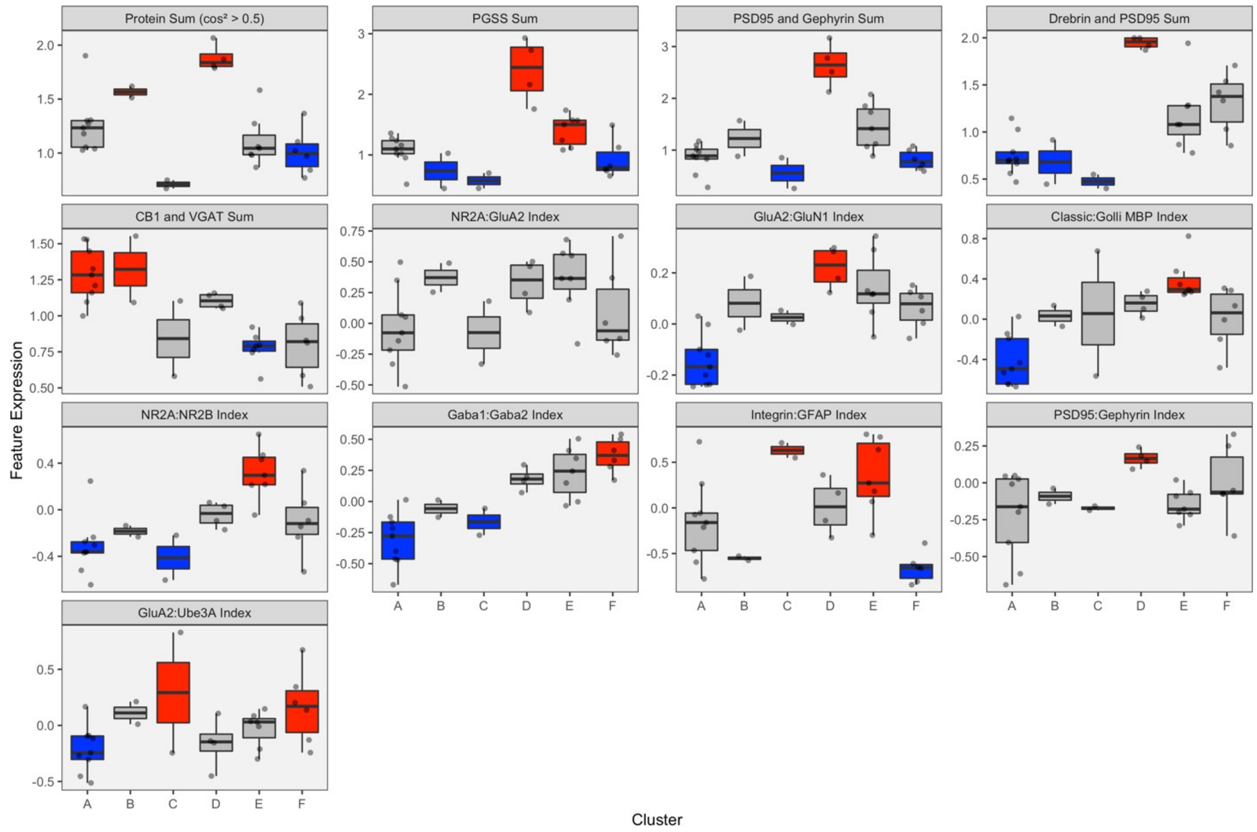

Supplementary Figure S7. *Boxplots show the expression of each feature within each cluster, with an alternate rule for identifying over- or under-representation*. Red coloured boxes represent the median expression of a group being greater than the upper quantile (i.e. 75th percentile) of all the data. Blue coloured boxes represent the median expression of a group being less than the bottom quantile (i.e. 25th percentile) of all the data. Boxes are grey if their median expression value is between the lower and upper quantiles.
